# Prevalence and Socioeconomic Factors of Diabetes and High Blood Pressure Among Women in Kenya: A Cross-Sectional Study

**DOI:** 10.1007/s44197-021-00004-6

**Published:** 2021-08-16

**Authors:** Sanni Yaya, Ziad El-Khatib, Bright Opoku Ahinkorah, Eugene Budu, Ghose Bishwajit

**Affiliations:** 1 School of International Development and Global Studies, University of Ottawa, Ottawa, ON Canada; 2 The George Institute for Global Health, The Imperial College London, London, UK; 3 Department of Global Public Health, Karolinska Institutet, 171 77 Stockholm, Sweden; 4 World Health Programme, Université du Québec en Abitibi-Témiscamingue (UQAT), Rouyn-Noranda, QC J9X 5E4 Canada; 5 School of Public Health, University of Technology Sydney, Sydney, Australia; 6 Department of Population and Health, University of Cape Coast, Cape Coast, Ghana

**Keywords:** Diabetes, High blood pressure, Non-communicable diseases, Sub-Saharan Africa, Global Health, Kenya

## Abstract

**Background:**

The emerging burden of high blood pressure (HBP) and diabetes in sub-Saharan Africa will create new challenges to health systems in African countries. There is a scarcity of studies that have reported associations of diabetes and HBP with socioeconomic factors on women within the population. We assessed the prevalence and socioeconomic factors of diabetes and high blood pressure among women in Kenya.

**Methods:**

We analysed cross-sectional data from the 2014 Kenya Demographic and Health Survey. Subjects were women aged 15–49 years. Self-reported status of HBP and diabetes was used to measure the prevalences. The association between educational and wealth index with HBP and diabetes was assessed by multivariable binary logistic regression.

**Results:**

The prevalences of self-reported HBP and diabetes were 9.4% and 1.3%, respectively. Women with secondary [aOR = 1.53; 95% CI = 1.15–2.02] and primary [aOR = 1.48; 95% CI = 1.15–1.92] levels of education were more likely to report having HBP, compared to those with no formal education. However, there was no significant association between educational level and self-reported diabetes. In terms of wealth quintile, we found that women with higher wealth quintile were more likely to report having HBP and diabetes compared to those with poorest wealth quintile. Specifically, the highest odds of self-reported HBP was found among women with richest wealth quintile compared to those with poorest wealth quintile [aOR = 2.22; 95% CI = 1.71–2.88]. Also, women with poorer wealth quintile were more likely to have self-reported diabetes compared to those with poorest wealth quintile [aOR = 1.89; 95% CI = 1.08–2.38].

**Conclusion:**

The prevalence of HBP and diabetes was low among women in Kenya. Household wealth status was associated with HBP and diabetes. No causation can be inferred from the data; hence, longitudinal studies focusing on health-related behaviour associated with NCDs are recommended. Proper dissemination of health information regarding the risk factors for HBP and diabetes may prove to be beneficial for NCD prevention programmes.

## Background

The emerging burden of non-communicable diseases (NCDs), particularly cardiovascular disease (CVD) and diabetes, threatens the gains in life expectancy made by combating infectious diseases [1, 2]. In the region of sub-Saharan Africa (SSA), where the majority of NCDs have long been considered “diseases of affluence”, CVD is becoming increasingly prevalent [3]. High blood pressure (HBP) is a common comorbidity for diabetes and up to 75% of adults living with diabetes also have HBP [4]. An increased likelihood of CVD occurs among individuals with diabetes and HBP [3]. The number of adults living with diabetes in SSA is estimated to increase more than twofold from 14.2 million people in 2015 to 34.2 million people in 2040 [5]. Complications from diabetes impose a substantial economic burden on individuals and the healthcare system [6, 7]. Furthermore, there has been an increase of 90% in HBP-related mortality during 1990–2015 [8].

Kenya is experiencing an urbanization growth, with 25% of Kenya’s population living in urban areas [9]. This includes the slums’ areas, known as the urban poor, which accounts for 56% of Kenya’s urban areas [10, 11]. This population suffers a double burden chronic of disease [12]. The severity of these diseases has been predicted to be likely greater than those of HIV and AIDS [3, 9]. Approaches to managing the overlapping risk factors for diabetes and HBP must emphasize modification of risk factors, such as lifestyle patterns [4]. There are insufficient data on the distribution of risk factors and outcomes in the SSA region to inform effective public health response [13].

The association of diabetes with disability differs across countries; however, diabetes has been shown to play a role in the disablement of people, thereby affecting their subjective health and well-being. Gender, age, education and level of income explain the association of diabetes with subjective health [14, 15]. Patients with medical comorbidities such as diabetes and HBP report poor subjective health [14]. Furthermore, gender is associated with the well-being of patients with diabetes and HBP [16, 17]. Women have been reported to be more likely to report poorer subjective health compared to men [15].

Comorbid conditions such as diabetes and HBP have been associated with obesity, which has been reported to be prevalent in women than men [18–21]. A contradictory finding from Seychelles showed that Socioeconomic status was inversely associated with the prevalence of diabetes among women [22]. Soubeiga et al. found, in Burkina Faso, the prevalence of hypertension in urban and rural areas to be 25% and 15%, respectively. Also, urban residents had higher levels of education and income when compared to rural residents [23]. A recent study has found that overweight and obesity are increasing among women of reproductive age in urban Africa, with obesity among this age group having more than doubled or tripled in 12 of the 24 countries [24]. This makes research on diabetes and HBP among women in Africa very critical.

In Kenya, a higher prevalence of hypertension among older obese women compared to men was observed in an urban slum [25, 26]. Furthermore, levels of awareness, treatment and control of diabetes and HBP among diabetic and hypertensive patients were generally low although women were more aware of diabetes and HBP than men [25, 26]. Shortage of screening opportunities, cost of treatment and preference for alternative medicine have been cited as reasons for low levels of awareness, treatment and control [27]. The early treatment of HBP in patients with diabetes can prevent CVD and the increased risk of morbidity and death [8, 25]. Another study reported a high prevalence of diabetes and HBP in Kenya due to lack of access to care and low public awareness [28]. Studies in the country have reported associations of diabetes and HBP with socioeconomic factors among the general population; yet, there is a lack of studies on women within the population. In order to strengthen the evidence about the magnitude of these health issues among women and further inform effective public health response, this study aims to assess the prevalence and socioeconomic factors of diabetes and HBP among women in Kenya.

## Methods

### Data Source

We used data from the 2014 Kenya Demographic and Health Surveys (DHS). The survey was conducted by the National Bureau of Statistics. This is a collaboration with other governmental and non-governmental entities, as part of the International Demographic and Health Survey programme known as Monitoring and Evaluation to Assess and Use Results (MEASURE) DHS, which is active in 90 countries and conducted under the auspices of the United States Agency for International Development (USAID) with the technical assistance of ICF International. This is a free, public dataset without any personal identifiers. We submitted a request to seek access to DHS data. This data request system ensures that all users understand and agree to basic data usage ethics standards. More information on the details of the survey and sampling procedures has been described by DHS [29]. The 2014 Kenya Demographic and Health Surveys (DHS) include 1612 clusters representing 617 in urban areas and 995 clusters in rural areas. Individual interviews were conducted to collect data on women of age 15–49 years and their children of under 5 years of age. In total, 14,728 women interviewed were included in the analysis.

### Variables Studied

#### Dependent Variables

Self-reported HBP and diabetes were the dependent variables in this study. The respondents were asked during interview whether they were told by doctor or health workers of having HBP and diabetes. The answers were coded as “yes” and “no”.

#### Explanatory Variables

The main explanatory variables were socioeconomic status which was measured by educational attainment and household wealth status. Educational level was categorized as: No education, Primary, Secondary and Higher. Wealth index in the DHS was assessed as an index of household assets and utilities using principal component analysis (PCA) and categorized as “poorest”, “poorer”, “middle”, “richer” and “richest”. The wealth index is calculated using easy-to-collect data on a household’s ownership of selected assets, such as televisions and bicycles, materials used for housing construction and types of water access and sanitation facilities. The calculation procedure of wealth index is available in the final report of Kenya Demographic and Health Survey 2014 [30].

#### Covariates

To adjust the analysis for potential confounders, the following covariates were included: age (15–19, 20–24, 25–29, 30–34, 35–39, 40–44, 45–49), place of residence (urban, rural), marital status (never in union, married, living with partner), religion (Christian, Muslim, other) and alcohol intake (no, yes).

### Data Analysis

Data were analysed using Stata software version 14.0. Three stages of analysis were done. The first step was the univariate analysis of all the variables used for the study (Table 1). Secondly, a bivariate analysis calculated the proportion of self-reported HBP and diabetes across the explanatory variables with their *p*-values which were derived from a Chi-square of fitness (Table 2). In the final step of the analysis, all variables which were significant at the Chi-square test were included in two hierarchical binary logistic regression models built for the outcome variables (Table 3). The variables that were significant at *p* < 0.25 were selected for inclusion in regression analysis. This was to make room for as many variables to be added to the regression analysis as possible. The first model, Model I, looked at a bivariate analysis between the explanatory variables and the outcome variables. Model II controlled for the effects of all the explanatory variables in a multivariable logistic regression (Table 3). Model I was the crude odds ratio (cOR), and Model II was the adjusted odds ratio (aOR). All frequency distributions were weighted, while the survey command (svy) in Stata was used to adjust for the complex sampling structure of the data in the regression analyses.

**Table 1:** Socio-demographic characteristics of the women in Kenya (*N* = 14,728)

Variables	Weighted *N*	Weighted %
*Educational level*		
No education	1020	6.9
Primary	7388	50.2
Secondary	4729	32.1
Higher	1590	10.8
*Wealth quintile*		
Poorest	2253	15.3
Poorer	2608	17.7
Middle	2878	19.5
Richer	3133	21.3
Richest	3855	26.2
*Age*		
15–19	2736	18.6
20–24	2710	18.4
25–29	2951	20.0
30–34	2177	14.8
35–39	1791	12.2
40–44	1301	8.8
45–49	1060	7.2
*Place of residence*		
Urban	5971	40.5
Rural	8757	59.5
*Marital status*		
Not married	4286	29.1
Married	8025	54.5
Cohabiting	745	5.1
Widowed	537	3.6
Divorced/separated	1135	7.7
*Religion*		
Christianity	13,513	91.7
Islam	921	6.3
Not religion	294	2.0
*Alcohol intake*		
No	14,027	95.2
Yes	701	4.8

**Table 2:** Proportions of self-reported HBP and diabetes across the socio-demographic characteristics

Variables	HBP (%) (9.4)	*p*-value	Diabetes (%) (1.3)	*p*-value
*Educational level*		< 0.001		0.060
No education	6.7		1.84	
Primary	9.3		0.9	
Secondary	9.7		1.6	
Higher	10.4		1.6	
*Wealth quintile*		< 0.001		0.084
Poorest	5.3		0.8	
Poorer	7.8		1.4	
Middle	8.6		1.1	
Richer	10.6		1.2	
Richer	12.3		1.7	
*Age*		< 0.001		< 0.001
15–19	2.7		0.5	
20–24	6.2		1.0	
25–29	9.8		1.4	
30–34	10.8		1.2	
35–39	12.0		1.3	
40–44	16.5		2.3	
45–49	17.4		2.6	
*Place of residence*		< 0.001		0.003
Urban	11.6		1.6	
Rural	7.8		1.0	
*Marital status*		< 0.001		0.047
Not married	4.5		0.9	
Married	11.0		1.4	
Cohabiting	10.1		1.1	
Widowed	11.3		0.9	
Divorced/separated	14.4		2.0	
*Religion*		0.001		0.063
Christianity	9.5		1.3	
Islam	7.8		1.7	
Not religion	9.2		0.3	
*Alcohol intake*		< 0.001		0.003
No	9.1		1.2	
Yes	13.5		1.9

**Table 3:** Logistic regression analysis on the effect of the socio-demographic characteristics of respondents on self-reported HBP and diabetes

Variables	HBP		Diabetes	
	Model I cOR (95% CI)	Model II aOR (95% CI)	Model I cOR (95% CI)	Model II aOR (95% CI)
*Educational level*				
No education	Reference (1.0)	Reference (1.0)	Reference (1.0)	Reference (1.0)
Primary	1.68*** (1.36–2.07)	1.48** (1.15–1.92)	0.64 (0.41–1.01)	0.76 (0.43–1.33)
Secondary	1.65*** (1.32–2.06)	1.53** (1.15–2.02)	0.91 (0.57–1.45)	1.21 (0.65–2.22)
Higher	2.09*** (1.60–2.73)	1.39 (1.00–1.94)	1.12 (0.62–2.01)	1.13 (0.52–2.43)
*Wealth quintile*				
Poorest	Reference (1.0)	Reference (1.0)	Reference (1.0)	Reference (1.0)
Poorer	1.76*** (1.42–2.18)	1.60*** (1.26–2.04)	1.53 (0.93–2.52)	1.89* (1.08–2.38)
Middle	2.04*** (1.66–2.52)	1.84*** (1.45–2.34)	1.11 (0.65–1.90)	1.29 (0.72–2.33)
Richer	2.36*** (1.92–2.89)	1.97*** (1.55–2.50)	1.29 (0.77–2.16)	1.21 (0.65–2.27)
Richest	2.76*** (2.26–3.37)	2.22*** (1.71–2.88)	1.84* (1.14–2.98)	1.37 (0.70–2.69)
*Age*				
15–19	Reference (1.0)	Reference (1.0)	Reference (1.0)	Reference (1.0)
20–24	2.48*** (1.83–3.34)	1.88*** (1.35–2.62)	2.27* (1.13–4.54)	2.13* (1.03–4.40)
25–29	4.00*** (3.02–5.28)	2.77*** (1.97–3.89)	2.35* (1.19–4.63)	2.25 (0.95–5.33)
30–34	4.29*** (3.22–5.72)	2.90*** (2.03–4.15)	2.85** (1.43–5.69)	2.84* (1.17–6.90)
35–39	4.85*** (3.64–6.47)	3.45*** (2.42–4.93)	2.82** (1.39–5.71)	2.90* (1.22–6.93)
40–44	8.03*** (6.03–10.68)	5.58*** (3.93–7.94)	5.14*** (2.62–10.11)	5.27*** (2.26–12.31)
45–49	8.00*** (5.97–10.73)	5.77*** (4.01–8.39)	4.72*** (2.33–9.57)	5.07*** (2.07–12.39)
*Place of residence*				
Urban	1.48*** (1.31–1.66)	1.24** (1.07–1.43)	1.56** (1.14–2.13)	1.40 (0.93–2.11)
Rural	Reference (1.0)	Reference (1.0)	Reference (1.0)	Reference (1.0)
*Marital status*				
Not married	0.35*** (0.29–0.42)	0.63*** (0.50–0.80)	0.58*** (0.38–0.87)	0.96 (0.54–1.71)
Married	Reference (1.0)	Reference (1.0)	Reference (1.0)	Reference (1.0)
Cohabiting	0.84 (0.63–1.13)	0.84 (0.62–1.13)	0.66 (0.27–1.63)	0.68 (0.27–1.69)
Widowed	1.22 (0.94–1.59)	0.98 (0.75–1.28)	0.83 (0.36–1.91)	0.66 (0.28–1.53)
Divorced/separated	1.22 (1.00–1.48)	1.07 (0.87–1.31)	1.27 (0.76–2.12)	1.10 (0.65–1.85)
*Religion*				
Christianity	Reference (1.0)	Reference (1.0)	Reference (1.0)	Reference (1.0)
Islam	0.69*** (0.57–0.84)	0.99 (0.79–1.24)	1.57* (1.06–2.33)	1.83* (1.13–2.95)
Not religion	0.97 (0.65–1.46)	1.32 (0.86–2.02)	0.63 (0.15–2.54)	0.63 (0.15–2.63)
*Alcohol intake*				
No	Reference (1.0)	Reference (1.0)	Reference (1.0)	Reference (1.0)
Yes	1.62*** (1.26–2.07)	1.33* (1.03–1.73)	2.26** (1.30–3.94)	2.08* (1.17–3.71)

*NB* Models II adjusted for age, place of residence, marital status, religion and alcohol consumption,* cOR *  crude odds ratio, *aOR* djusted odds ratio, *CI* confidence interval

*p < 0.05, **p < 0.01, ***p < 0.001

## Results

### Descriptive Results of Socio-demographic Characteristics of Women in Kenya

In total, 14,728 women aged between 15 and 49 years were included in the study (Table 1). Of them, one-fifth (20%) were of 25–29 years of age, more than half (59.5%) were rural residents and 54.5% were married. Rate of alcohol consumption was 4.8%. Rate of literacy was 83.1%, with more than half of the women having primary level qualification (50.2%), and 10.8% higher than secondary education. Regarding wealth status, more than a quarter were living in the richer households (26.2%) and 91.7% were Christians.

### Prevalence of Self-Reported HBP and Diabetes by the Socio-demographic Characteristics of Women in Kenya

The prevalence of HBP and diabetes in Kenya was 9.4% and 1.3%, respectively, with variations across the socioeconomic and demographic variables (Table 2). At a *p* < 0.25, all the explanatory variables and covariates had significant associations with self-reported HBP and diabetes.

### Socioeconomic Predictors of Self-Reported HBP and Diabetes

We found that women with secondary [aOR = 1.53; 95% CI = 1.15–2.02] and primary [aOR = 1.48; 95% CI = 1.15–1.92] levels of education were more likely to report having HBP, compared to those with no formal education. However, having higher education had no significant association with HBP. Also, there was no significant association between educational level and self-reported diabetes. In terms of wealth quintile, we found that all levels of wealth quintile were statistically significant for HBP, but only poorer versus poorest was significant for diabetes. Specifically, women with richest [aOR = 2.22; 95% CI = 1.71–2.88], richer [aOR = 1.97; 95% CI = 1.55–2.50], middle [aOR = 1.84; 95% CI = 1.45–2.34] and poorer [aOR = 1.60; 95% CI = 1.26–2.04] wealth quintile were more likely to report having HBP compared to those with poorest wealth quintile. We also found that women with poorer wealth quintile were more likely to have self-reported diabetes compared to those with poorest wealth quintile [aOR = 1.89; 95% CI = 1.08–2.38] (Table 3, Model II).

## Discussion

In order to strengthen the evidence about the magnitude of HBP and diabetes among women and further inform effective public health response, the present study assessed the prevalence and socioeconomic factors of diabetes and high blood pressure among women in Kenya. The results indicated that the prevalence of HBP was higher than that of diabetes (9.4% vs 1.3%). The rates are comparatively lower than global averages—24.8% for females (2012 estimate) [31] and 2.8% regardless of sex (2000 estimate) [32]. The difference can be explained in part by the level of living standards and distribution of risk factors such as dietary factors and physical activity. For instance, developed countries or countries with higher level of urbanization, better transportation facilities and employment in the service sector are likely to have lower level of physical activity compared to those where the population is predominantly rural with agriculture being the primary source of livelihood. The prevalence of HBP in Kenya was also found to be lower in a cross-sectional study covering four countries, which reported that the prevalence was 21.4% in rural Kenya (23.7% in urban Tanzania and 38.0% in urban Namibia) [33]. The current prevalence shows a reduction in prevalence of diabetes between 2009 and 2014. The low prevalence of diabetes in Kenya could be due to the general eating habits of women, defined by low consumption of sugary foods and living conditions that are associated with some levels of physical activity. Overall, variations in prevalence rates of HBP and diabetes may also be due to the limited age range, method of estimation and data collection. In the KDHS survey, participants were asked if they were ever told by a physician whether or not they have the disease. There remains the possibility of underreporting/under diagnosis as not all the women might have undergone a diagnosis. Therefore, the findings may not represent the exact situation of HBP and diabetes among women in the country.

The findings from the multivariable analysis suggested a positive association between self-reported HBP and diabetes and wealth status. Women in the richest wealth quintiles and those in the poorer wealth quintiles were found to have higher odds of HBP and diabetes compared with those in the lowest wealth quintile (poorest). Socioeconomic disparities have been the subject of intense research in the context of health-related outcomes. In general, socioeconomically disadvantaged communities suffer more unhealthy conditions such as poor hygiene, improper diet, environmental and psychosocial stress, which alone or in combination with other factors increases the likelihood of developing certain disease conditions [34–37]. However, the impact of one’s socioeconomic position on health status may not always be as straightforward, especially when it comes to lifestyle-related behaviours. For example, households with improving economic status are more likely to afford meat and other animal-sourced food products [38] and consume lesser proportion of fruits and vegetables, which can potentially lead to developing metabolic syndromes such as higher blood pressure and insulin resistance [39]. Other possible reasons for this finding could be that patients with higher wealth quintile could have a higher level of cultural sensitization leading them to be more aware of their health status [40, 41]. Women with poorest wealth quintile may be unaware of their health status, including symptoms of HBP and diabetes, and this is likely to affect their experience and treatment for HBP and diabetes. Other possible reasons for this finding are that richer women are more likely to deal with barriers to healthcare access including visits to the health facilities, distance to the health facilities and financial cost of healthcare access [42–44]. This implies that they are more likely to have easy access to healthcare facilities and hence report HBP and diabetes if they are diagnosed of it.

We also found that women with higher level of education had higher odds of reporting HBP. However, there was no significant association between level of education and diabetes. Contrary to our findings on the positive association between level of education and self-reported HBP, previous research suggests that level of education has an inverse relationship with blood pressure and risk of hypertension [45, 46], even after adjusting for income and other measures of socioeconomic status [47]. However, education is typically characterized using only years of schooling or degree attainment, but not both. Years of schooling and degree attainment differ importantly in their conceptualization of the underlying mechanisms linking education to health [48]. This may account for the inverse relationship between level of education and self-reported HBP in previous studies. However, in relation to our findings on the direct relationship between level of education and self-reported HBP, the possible reason could be that women with higher levels of education are more likely to have higher wealth quintile and hence can afford meat and other animal-sourced food products [38]. Despite this, educated women are also more likely to seek health care, compared to non-educated women [43], and hence are more exposed to the risk of getting diagnosed of HBP. Just like women with richest wealth quintile, educated women are also likely to overcome some of the barriers to healthcare services [42–44], making them more likely to be diagnosed of HBP. Most of them are also more likely to have a higher level of cultural sensitization leading them to be more aware of their health status [40, 41].

### Practical Implications of the Findings

Changing demographics, urbanization, dietary and lifestyle behaviour are contributing to the rising prevalence of NCDs, e.g. diabetes and HBP. In order to make informed policy decisions to address the factors that aggravate the situation, it is necessary to have country-level information on the burden of the diseases. While the behavioural components of individual socioeconomic status may be hard to address, efficient dissemination of health knowledge regarding the risk factors for NCDs through mass media can have promising outcomes. According to our findings, it is suggestible that women with higher wealth status are more likely to live with HBP and diabetes, while those with higher level of education are more likely to report having HBP and require attention from health researchers to devise appropriate policy solutions. In the light of the present analysis, we cannot precise the pathways through which wealth status affect blood pressure status as there was no information on health behaviour. To the best of our knowledge, currently there is no country-wide survey in Kenya on health-related behaviour and its impact on health outcomes. Further studies are therefore recommended to explore the mediating role of health behaviour in the association between wealth status, and HBP and diabetes. Though the prevalence of diabetes was considerably low, the possibility of further expansion remains high given the higher prevalence of HBP, which is an important risk factor for diabetes.

### Strengths and Limitations

This study has several strengths and limitations to report. The data were collected from DHS survey which is a renowned source of population health-related information in developing countries. Sample size was considerably large and representative of Kenyan women ageing 15–49 years. As far as we are concerned, the current literature on the prevalence of risk factors for HBP and diabetes is very limited for Kenya and other countries in the region. Hence, the findings are expected to be valuable to health researchers and health policy practitioners. However, the findings need to be interpreted with caution since the disease status was self-reported and was not verified by objective measurements, which carries the chance of underreporting. The data were cross sectional; therefore, the association cannot be regarded as causation between the variables. Finally, this study did not examine interaction effects between variables in the logistic regression analysis, and this may limit the robustness of the analysis.

## Conclusion

The findings conclude that the prevalence of HBP and diabetes is considerably lower among women in Kenya. Household wealth status appeared to be associated with HBP and diabetes. However, educational status showed significant association with HBP, but not with diabetes. No causation can be inferred from the data; hence, longitudinal studies focusing on health-related behaviour associated with non-communicable diseases (NCDs) are recommended. Proper dissemination of health information regarding the risk factors for HBP and diabetes may prove to be beneficial for NCD prevention programmes in the country.

## Data Availability

The data that support the findings of this study are openly available and can be accessed on http://dhsprogram.com/methodology/survey/survey-display-566.cfm.

## Abbreviations

CVD: Cardiovascular diseases
HBP: High blood pressure
NCDs: Non-communicable diseases
SSA: Sub-Saharan Africa (SSA)
SES: Socioeconomic status
